# Promyelocytic sarcoma of the right humerus: an unusual clinical presentation with unique diagnostic and treatment considerations

**DOI:** 10.1002/ccr3.1212

**Published:** 2017-09-29

**Authors:** Sameer Sawhney, Noa G. Holtzman, Derik L. Davis, Hannah Kaizer, Victoria Giffi, Ashkan Emadi, Rima Koka

**Affiliations:** ^1^ Department of Pathology University of Maryland School of Medicine Baltimore Maryland; ^2^ University of Maryland Greenebaum Comprehensive Cancer Center Baltimore Maryland; ^3^ Department of Medicine University of Maryland School of Medicine Baltimore Maryland; ^4^ Department of Diagnostic Radiology & Nuclear Medicine University of Maryland School of Medicine Baltimore Maryland; ^5^ University of Maryland School of Medicine Baltimore Maryland; ^6^ Meritus Medical Center Hagerstown Maryland

**Keywords:** Acute promyelocytic leukemia, leukemia, promyelocytic Sarcoma

## Abstract

Promyelocytic leukemia is a known medical emergency and requires rapid diagnosis and expedient therapy with differentiating agents. We present an unusual case in which the diagnosis is based on a fine needle aspirate of a humeral mass. Despite lack of systemic involvement, the sarcoma responded to traditional differentiation agents.

## Introduction

Acute promyelocytic leukemia (APL) is defined by the World Health Organization (WHO) as a subtype of acute myeloid leukemia (AML) with recurrent cytogenetic abnormalities, typically demonstrating t(15;17)(q22q21) involving the fusion of the promyelocytic leukemia gene (PML) and the retinoic acid receptor‐alpha gene (RARA). Patients often present with disseminated intravascular coagulopathy (DIC) with leukocytosis or leukopenia. It is imperative that this diagnosis be histologically and cytogenetically confirmed so that differentiation therapy with all‐trans retinoic acid (ATRA) and arsenic trioxide can be promptly started. Although these patients usually have excellent clinical outcomes if treated expeditiously, unusual clinical presentations may cause delayed diagnosis and treatment. In this report, we present a patient with promyelocytic sarcoma with no peripheral blood or iliac crest bone marrow involvement by leukemia. The diagnostic challenge, treatment, and clinical course are of particular interest given the unique clinical circumstances.

## Case Presentation

**Figure 1:**
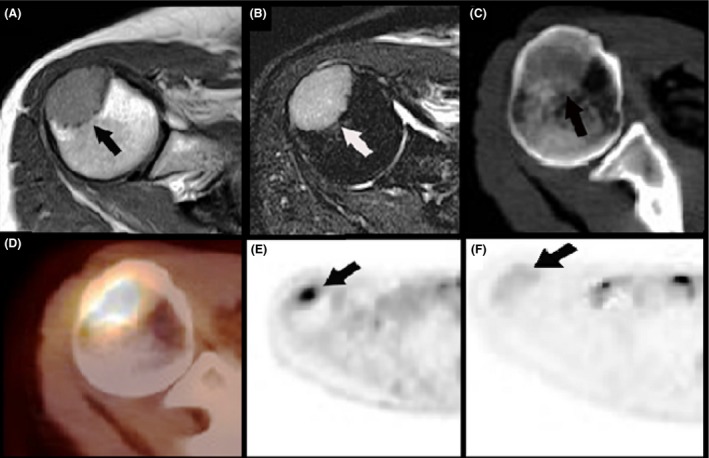
(A) Axial T1‐weighted MR image shows an intramedullary bone mass (arrow) with intermediate signal in the greater tuberosity of the proximal right humerus. (B) Axial T2‐weighted fat‐saturated MR image shows corresponding T2‐bright signal in the mass (arrow). (C) Axial unenhanced CT image shows an osteosclerotic intramedullary bone mass (arrow) in the proximal right humerus. (D) ^18^F‐FDG PET/CT fused image shows increased FDG avidity in the mass with an SUV of 7.4. (E) ^18^F‐FDG PET image shows a FDG‐avid intramedullary bone mass (arrow) in the proximal right humerus before treatment. (F) ^18^F‐FDG image following treatment 1 month later shows interval resolution of metabolic activity (arrow).

A 52‐year‐old woman was seen in the outpatient orthopedics clinic for evaluation of right upper extremity swelling and pain of 3‐week duration (pain score of six at its worst), which initially extended from her right shoulder down to the fingers, and resolved without treatment, albeit with residual pain at presentation. The patient reported subjective fevers that occurred at the onset of swelling and spontaneously resolved within 24 h. Physical examination of the right shoulder revealed full active and passive range of motion with mild tenderness over the greater tuberosity of the right humerus. Laboratory tests including complete blood count with differential, and serum protein electrophoresis were unremarkable except for elevated erythrocyte sedimentation rate (ESR) and C‐reactive protein (CRP). Magnetic resonance imaging (MRI) of the right shoulder demonstrated a nonspecific intramedullary mass in the greater tuberosity of the proximal humerus, measuring 3.2 × 2.1 × 2.8 cm (Fig. 1A and B). Unenhanced computed tomography (CT) showed an osteosclerotic intramedullary mass, with a corresponding SUV of 7.4 on 18‐fluorodeoxyglucose positron emission tomography (PET) (Fig. 1C and D).

**Figure 2:**
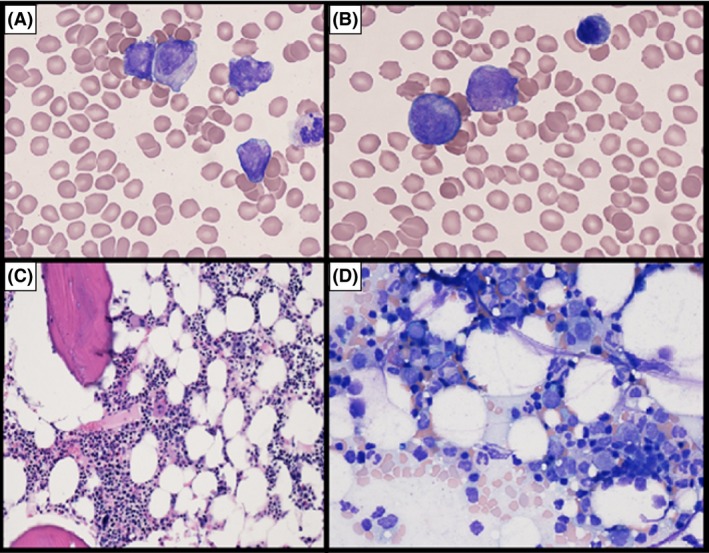
(A) Atypical promyelocytes with identifiable auer rods from right humerus (B)Atypical promyelocytes with bilobed nuclei from right humerus (C) 40x H&E section of bone marrow core biopsy revealing normocellular bone marrow (40%) for patients age without evidence of atypical promyelocytes. (D) Wright Giemsa prepared bone marrow aspirate showing erythroid predominance without evidence of atypical promyelocytes.

Core needle biopsy and cultures of the right proximal humerus lesion were performed (Fig. 2A and B). The aspirate demonstrated numerous atypical promyelocytes with convoluted nuclear contours and abundant cytoplasm with large granules. A few cells with numerous Auer rods were also present. Flow cytometry revealed that these promyelocytes expressed CD33, CD117, CD71, cytoplasmic MPO and CD34 (partial), but were negative for HLA‐DR, CD11c, and CD11b. Fluorescence In situ‐Hybridization (FISH) was promptly performed on the aspirate, and identified a t(15;17) translocation with fusion of PML/RARA in 79% of interphase cells.

A bone marrow biopsy and aspirate from the patient's right iliac crest were subsequently performed to evaluate for systemic disease (Fig. 2C and D). Interestingly, neither the peripheral blood nor the bone marrow of the iliac crest showed morphologic or cytogenetic evidence of promyelocytic leukemia.

FISH studies were negative for t(15;17), and RT‐PCR was negative for the PML/RARA fusion in both the peripheral blood and bone marrow specimens. These findings confirmed that the patient had promyelocytic sarcoma involving the right humerus but without the typical leukemic presentation of APL.

Additional CT scans performed for staging revealed abnormally increased metabolic activity in the sternum and manubrium, acromioclavicular joints, left acromium, right humeral head with mild increased osseous density, bilateral humeral diaphysis, bilateral proximal ulnar and radial heads, bilateral distal femurs, right distal femoral metaphysis, and bilateral proximal tibias (SUVs ranging from 7.4 to 27.4). No other abnormalities were noted.

The patient was started on treatment as per Lo‐Coco et al. with all‐trans retinoic acid (ATRA) and arsenic trioxide which she tolerated well without any complications 1. The overall clinical course from the time of onset of pain to initiation of differentiating agents was 4 weeks. Disease response was monitored by PET/CT evaluation which was repeated at day 30 of treatment and showed incomplete response, with persistent asymmetric increased metabolic activity in the bone marrow of the distal right femur and proximal right tibia, compatible with residual leukemia. The previously described diffuse metabolic activity in the remainder of the osseous and bone marrow structures including the right humerus (Fig. 1F), sternum, manubrium, and acromioclavicular joints was no longer present. After completing induction, treatment was held for 2 weeks. Patient was then continued onto consolidation therapy with ATRA and arsenic, consisting of four 8‐week cycles for a total 28 weeks of consolidation, which she tolerated well without complications. Our patient exhibited an excellent treatment response, with PET/CT scan after completion of consolidation therapy showing a complete resolution of all previously seen abnormal metabolic activity, with no new metabolically active lesions. She is planned to proceed with maintenance therapy with 2 weeks of ATRA alone given every 3 months for the next year.

## Discussion

Herein, we describe the case of a patient diagnosed with APL with a highly unusual presentation. Neither the peripheral blood nor the bone marrow aspirated from the iliac crest was positive for APL characteristic gene rearrangement, or had the morphologic features of APL. The aspirate from the right humeral mass, however, was positive for these APL‐defining features, thus resulting in a diagnosis of primary promyelocytic sarcoma.

Acute promyelocytic leukemia rarely presents as a mass‐forming lesion without evidence of leukemic involvement, thus making this a highly unique case. Only a few cases have been reported in primary APL presents as a solitary lesion 2, 3, 4. Although rare, it appears more common in cases with relapsed APL to present as an extramedullary mass compared to primary disease 5, 6, 7, 8. AML can manifest as a soft tissue tumor with or without concurrent marrow findings of AML, and these cases are designated as a myeloid sarcoma by the WHO 8. The most common subtype to present in such a way is acute monoblastic leukemia.

Isolated myeloid sarcoma has often been misdiagnosed as a malignant lymphoproliferative disorder, Ewing sarcoma, or other hematopoietic neoplasm, often due to inadequate immunophenotyping of the malignancy 8, 9. The case presented herein illustrates a potential diagnostic pitfall and the importance of prompt morphologic evaluation, as well as thorough immunophenotyping and cytogenetic analysis. Flow cytometry, in addition to FISH and metaphase cytogenetics, are indispensable tools to differentiate APL from malignant lymphoproliferative disorders, sarcomas, and even other myeloid sarcomas. This is particularly important in the case of APL, as it is a medical emergency, and an improper diagnosis may delay time‐sensitive life‐saving measures.

The unusual presentation also raises questions regarding treatment and prognosis of such patients. It is unclear whether the same propensity for DIC exists in patients who lack leukemic manifestation of the disease. Additionally, it would of interest to determine if there are additional genetic or epigenetic changes within the leukemic cells which allowed for such an unusual presentation. At present, it is of great importance that the patient has responded well to traditional APL regimens as assessed by imaging studies.

## Authorship

SS: finalized the writing of the manuscript with an emphasis on the pathologic elements and integrating radiographic images into the report. NH: finalized the writing of the clinical portion of the manuscript. DLD: contributed the radiographic images, contributed to interpretation, and helped edit the manuscript to reflect radiographic findings. HK: wrote the early draft of clinical history. VG: contributed to the clinical portion of the manuscript. AE: treated the patient and was involved in final manuscript editing with Rima Koka. RK: diagnosed the patient and was involved in final manuscript editing with Ashkan Emadi.

## Conflict of Interest

None declared.
